# Atopic March or Atopic Multimorbidity—Overview of Current Research

**DOI:** 10.3390/medicina60010021

**Published:** 2023-12-22

**Authors:** Iva Mrkić Kobal, Davor Plavec, Željka Vlašić Lončarić, Ivana Jerković, Mirjana Turkalj

**Affiliations:** 1 Clinic for Pediatric Medicine Helena, Ulica kneza Branimira 71, 10000 Zagreb, Croatia; 2 Faculty of Medicine, Josip Juraj Strossmayer University of Osijek, Josipa Huttlera 4, 31000 Osijek, Croatia; 3 Prima Nova, Zagrebačka cesta 132a, 10000 Zagreb, Croatia; 4 Children’s Hospital Srebrnjak, Srebrnjak 100, 10000 Zagreb, Croatia; 5 Faculty of Medicine, Catholic University of Croatia, Ilica 242, 10000 Zagreb, Croatia

**Keywords:** atopic march, atopic dermatitis, allergic rhinitis, allergic asthma, pathological mechanism, risk factors, prevention

## Abstract

The atopic march encompasses a sequence of allergic conditions, including atopic dermatitis, food allergy, allergic rhinitis, and asthma, that frequently develop in a sequential pattern within the same individual. It was introduced as a conceptual framework aimed at elucidating the developmental trajectory of allergic conditions during childhood. Following the introduction of this concept, it was initially believed that the atopic march represented the sole and definitive trajectory of the development of allergic diseases. However, this perspective evolved with the emergence of new longitudinal studies, which revealed that the evolution of allergic diseases is far more intricate. It involves numerous immunological pathological mechanisms and may not align entirely with the traditional concept of the atopic march. The objective of our review is to portray the atopic march alongside other patterns in the development of childhood allergic diseases, with a specific emphasis on the potential for a personalized approach to the prevention, diagnosis, and treatment of atopic conditions.

## 1. Introduction

The atopic march is a well-known concept that describes the natural progression of allergic diseases in individuals, particularly during childhood. It refers to the sequential development of different allergic conditions in a predictable pattern within the same person [1]. This concept framework is rooted in insights driven by epidemiological studies [2]. Typically, the atopic march begins with atopic dermatitis (AD) in infancy, followed by the development of allergic asthma and allergic rhinitis as the individual grows older [1].

Atopic dermatitis emerges in 17–24% of children [3]. and 10% of adults worldwide [4]. Among children with mild atopic dermatitis, the prevalence of asthma is approximately 20%, but it escalates to over 60% in those with severe atopic dermatitis [5]. In children diagnosed with asthma (AA), approximately 74–81% also experience allergic rhinitis (AR) [6], and these children typically show signs of allergic rhinitis at an earlier age (2.9 ± 1.7 years) [7].

In the birth cohort study focused on the Multicenter Allergen Study (MAS), Gough et al. have illuminated a noteworthy uptick in the prevalence of AD, asthma, and allergic rhinitis among children with a positive family history of atopy [8]. Similarly, findings from the Dampness in Building and Health (DBH) study by von Kobyletzki et al. have demonstrated that children afflicted with AD exhibit significantly elevated odds ratio (OR) of developing allergic asthma (AA) (OR 3.07; 95% confidence interval (CI) 1.79–5.27) and allergic rhinitis (AR) (adjusted OR 2.63; 1.85–3.73) during follow-up, in contrast to their counterparts without AD [9]. Furthermore, the results of the 2018 Canadian Healthy Infant Longitudinal Development (CHILD) study conducted in Canada have solidified the notion of an augmented risk of AA (absolute risk reduction (aRR), 2.23; 95% CI 1.36–3.67) and AR (aRR 4.44; 95% CI 2.59–7.63) in three-year-old children previously diagnosed with AD [10]. Concurrently, the PASTURE birth cohort study has also brought to light an escalated risk of AA in children by the age of six who had early and persistent AD (adjusted OR 2.87, 95% CI 1.31–6.31) [11]. All these studies, along with numerous others, indicated a propensity for the atopic march, suggesting a natural progression of atopic diseases in individuals [8,9,10,11,12,13,14,15].

The emergence of machine learning has led to a reevaluation of the atopic pattern. [16]. In 2014, Belgrave and colleagues presented findings from two population-based cohort studies that delved into the individual profiles of eczema, wheezing, and rhinitis. Their research also sought to determine if these symptoms adhered to an atopic march pattern in their onset. Their investigations confirmed the heterogeneous nature of developmental profiles for eczema, wheezing, and rhinitis. It was revealed that only a minor fraction of children (7% of those experiencing symptoms) exhibited trajectory profiles that resembled the atopic march [17].

Our review endeavors to provide a comprehensive understanding of the diverse trajectories involved in the development of atopic diseases, with a particular focus on demystifying the concept of the atopic march and assessing its presence within specific populations. Our objectives encompass the assessment of risk factors associated with the progression of the atopic march, alongside an exploration of diagnostic and therapeutic strategies grounded in personalized medicine.

We conducted an extensive exploration of scholarly literature in English within the Web of Science and PubMed databases, using keywords such as “atopic dermatitis,” “atopic march,” “allergic asthma,” and “rhinitis” within the timeframe spanning from 2017 to 2023.
**Keypoints**The atopic march is a concept representing natural development of atopic diseases, from atopic dermatitis to allergic asthma and rhinitisAtopic march occurs in only 3.1% of children with atopic diseaseThe newer studies emphasize the atopic multimorbidity concept rather than atopic marchIt is essential to improve our understanding of the physiological mechanisms and risk factors to accurately identify individuals with atopic disease progression and implement effective preventive measures.

## 2. Concurrent and Overlapping Atopic Disease: A Multimorbidity Exploration

The development of different atopic diseases as well as their overlapping is still a subject of research. The way symptoms manifest across various organs or systems, such as the skin, lungs, and nasal passages, can offer insights into the underlying pathophysiological processes. However, there is substantial diversity in the timing of symptom initiation and progression among individuals. Understanding pathophysiological mechanisms could offer a mode of intervention for disease prevention as well as treatment [18].

The advent of machine learning has opened the door to the potential for disentangling atopic phenotypes through the analysis of clinical symptom patterns, facilitating discussions about the co-occurrence and concurrent presence of atopic diseases. Bayesian approaches appear particularly well-suited for this endeavor. In layman’s terms, Bayes’ theorem serves as a tool for guiding us to modify our convictions when confronted with fresh evidence [19,20].

Belgrave and colleagues employed a Bayesian approach to model the progression of allergic conditions, including eczema, wheezing, and rhinitis, throughout the childhood of over 10,000 children drawn from two United Kingdom (UK) population-based birth cohorts. Their primary emphasis was on tracking longitudinal shifts within individual children. They evaluated three distinct models concerning the symptoms and diseases under investigation. The initial model postulated that eczema, wheezing, and rhinitis each exhibit independent profiles over time. It suggested that the transition of acquiring, persisting, or remitting each disease followed a sequence of independent events, where the likelihood of each disease’s occurrence depends solely on its previous state (i.e., eczema influencing eczema, wheezing affecting wheezing, and rhinitis influencing rhinitis). The second model assessed a comorbidity framework rooted in the concept of the “atopic march” prior to considering any data analysis. It was assumed that eczema serves as the index condition, affecting subsequent predisposition to wheezing, which in turn predicts the later onset of rhinitis. Nevertheless, both models failed to achieve consistent convergence, suggesting that the data did not substantiate the assumptions that either eczema, wheezing, and rhinitis are independent of each other or that they adhere to a sequential “atopic march” framework [17].

In the third model, it was postulated that a multimorbidity framework should be considered, where no individual condition takes precedence. Instead, the combined presentation of eczema, wheezing, and rhinitis in each child could be elucidated by their association with a specific hidden disease class profile.

They have found 7 distinct atopic disease classes: (1) atopic march (3.1%), (2) persistent eczema and wheeze (2.7%), (3) persistent eczema with later-onset rhinitis (4.7%), (4) persistent wheeze and later-onset rhinitis (5.7%), (5) wheeze only—transient (7.7%), (6) eczema only (15.3%), and 7% rhinitis only (9.6%). The other 51.2% of children included in the study had no atopic disease [17,19].

The models, when subjected to data analysis, unmistakably revealed that a solitary, linear, cause-and-effect model cannot encapsulate the diversity inherent in allergic phenotypes [17]. This research underscores that the notion of a single, linear progression is antiquated and of limited utility in understanding these conditions. Nonetheless, the absence of a direct link between eczema, wheezing, and rhinitis does not imply that there is no association between them [21]. On the contrary, even a model assuming independence among these conditions failed to provide a satisfactory explanation for the data [22]. These conditions frequently co-occur more frequently than would be expected by random chance. However, this coexistence likely occurs within a framework of multimorbidity, wherein “no single condition takes precedence over any of the concurrent conditions in the eyes of the patient and healthcare professionals” [23].

It’s important to emphasize that the identification of concurrent presence, comorbidity, or multimorbidity among eczema, asthma, and rhinitis should not be misconstrued as evidence of any interconnection among them, let alone a causal one.

This implicates the need for individual disease endotyping and an individual approach to disease diagnosis and treatment [24].

## 3. Atopic Dermatitis

Atopic dermatitis is a chronic relapsing skin disorder characterized by skin itching, inflamed skin patches, skin barrier dysfunction, eosinophilic skin inflammation and recurrent skin infections. It has high impact on quality of life [25]. The prevalence of AD worldwide according to International Study of Asthma and Allergies in Childhood (ISAAC) phase III is 7.9% in 6-to 7- years old and 7.3% in 13-to 14 years old [26].

In numerous children, atopic dermatitis serves as the initial indication of an atopic predisposition, often manifesting in early infancy [27].

It presents itself as a intricate condition, characterized by a variety of contributing factors. The roots of this complexity lie in the dysregulation of the immune system and the dysfunction of the epidermal barrier, both of which are impacted by a combination of genetic predisposition and environmental influences [28]. As the atopic march delineates a natural sequence of atopic symptoms evolving from AD to asthma and AR, a prevailing notion has emerged. This perspective implies that AD tends to initiate a sequence that may lead to the development of other allergic conditions. Many pediatric patients with atopic dermatitis develop other atopic disease or eventually overcome the disease [29].

The advent of recent longitudinal studies employing advanced statistical methods has unveiled distinct clusters within atopic dermatitis. This development poses a challenge in accurately identifying the risk group for the subsequent development of atopic diseases [30].

Nevertheless, the data, encompassing information from the cohorts considered in this review, indicate associations between the risk of progression and various factors. These include a younger age of onset, a family history of atopy, heightened severity of AD, filaggrin mutations, an urban environment, and polysensitization and/or allergic comorbidities. Two of the most frequently identified risk factors for the persistence of AD are the early onset of the disease and its severity during infancy [31]. The PASTURE cohort has indicated an elevated risk of asthma development in children exhibiting an early persistent phenotype [11]. Similarly, the Tucson Children Respiratory Study identified AD in infancy as an independent risk factor for persistent wheezing [32]. Within the Pediatric Eczema Elective Registry (PEER) cohort, where 73% of patients reported AD before the age of 2, there is a significantly higher risk of developing new seasonal allergies. Positive family history has also been confirmed as an independent risk factor for development of AD, as well as for persistence of other atopic conditions such as asthma [33]. In PASTURE’s study participants with both parents having a history of allergy had approx. sixfold higher risk of the early persistent AD trajectory [11].

Clinicians generally concur that individuals with more severe AD are prone to exhibit both multimorbidity and a more persistent course of the disease [34]. These was also confirmed in MAS cohort study where severity of AD had been shown to be the strongest risk factor for poor prognosis [3]. The BAMSE cohort had also shown that the AD severity was significantly related to asthma onset [35].

## 4. Underlying the Atopic March/Atopic Multimorbidity: Patological Mechanism

Considering the various multimorbid disease trajectories explored earlier, it is probable that several unique pathways and mechanisms are at play. Some of these may be shared across all atopic/T helper (TH) 2-dominant diseases, while others could be specific to diseases [36]. How is it that AD contributes to the development of asthma? Many inquiries into the causal relationship labeled as “progression” focus on finding evidence supporting the idea that early childhood AD fosters the emergence of food allergies (FA) and respiratory allergies. This is believed to occur through systemic sensitization, a consequence of compromised skin barrier function. Consequently, the hypothesis attributing the primary cause of atopic diseases to a deficiency in epithelial barrier integrity is endorsed [37,38].

Skin barrier predominantly depends on stratum corneum. The core of the corneocytes is primarily composed of keratin filaments organized by filaggrin (FLG), a key component that forms a framework for the extracellular lipid matrix [39]. The absence of properly functioning FLG, attributed to FLG gene mutations and the impact of inflammatory and pro-inflammatory mediators affecting FLG expression (acquired deficiency), leads to a disruption in the essential processes required for the stratum corneum’s protective role [40]. In particular, the compromised function of the damaged skin barrier facilitates the entry of diverse allergens/haptens, environmental pollutants, and toxins [41]. Functional loss resulting from mutations in FLG, which specifically impact the epidermal barrier, increases the susceptibility to peanut allergy. This association holds true even when accounting for the presence of AD and employing varying levels of stringency in defining peanut allergy [42,43,44]. Notably, a heightened exposure to environmental peanut allergens, coupled with both FLG mutations and the presence of AD, amplifies the risk of developing peanut allergy in an additive manner [45].

Thymic stromal lymphopoietin (TSLP) is a cytokine that belongs to 4-helix bundle cytokine family, primarily targeting dendritic cells. In co-culture, these dendritic cells have the capacity to stimulate the production of IL-4, IL-5, and IL-13 from naive CD4+ T cells [46]. TSLP is naturally expressed at baseline levels in mucosal surfaces such as the gut and lung, as well as in the skin. Moreover, its expression can be heightened in response to exposure to viral, bacterial, or parasitic pathogens, as well as Toll-like receptor (TLR) agonists [47]. Apart from influencing Th2 cell polarization via antigen-presenting cells, TSLP also has a direct impact on CD4+ T cells, CD8+ T cells, and Treg cells [48,49]. TSLP’s reach extends to promoting Th2 cytokine responses by affecting mast cells, innate lymphoid cells (ILCs), epithelial cells, macrophages, and basophils. Notably, TSLP plays a significant role in basophil biology; in vitro, it can induce basophil maturation from bone marrow precursors in an IL-3 independent manner [50]. Additionally, basophils elicited by TSLP in vivo exhibit a distinct phenotype compared to those elicited by IL-3 [50,51].

The connection between atopic diseases and TSLP expression was initially established by Soumelis et al., who observed significantly increased expression in the lesion skin of individuals with AD [52]. Subsequent investigations revealed TSLP expression in the airways of asthmatics and the nasal passages of individuals with AR [50,53]. In asthmatic airways, TSLP levels correlated with the expression of Th2-attracting chemokines and the severity of the disease [54].

Our full comprehension of the mechanisms by which AD may predispose an individual to asthma in children population remains incomplete. The connection found in diverse studies have also raised the hypothesis of decreased antiviral interferon production in children with AD due to AD related cytokines [18].

Children exhibiting compromised anti-viral interferon production face a scenario where virus infected nasal epithelial cells discharge viruses (for example rhinovirus or respiratory syncytial virus) into lower respiratory tract. That induces necrosing cell death that release active interleukin-33, ultimately triggering type 2 inflammation, and wheeze [55]. Additionally, antiviral interferons play a crucial role in directly inhibiting proliferation of Th 2 cells and type 2 innate lymphoid cell, as well as the production of type 2 cytokines, IL-4, IL-13 [56]. One critical factor contributing to compromised antiviral interferon (IFN) production involves the interconnection of IgE molecules present on the surface of plasmacytoid dendritic cells (pDCs). These cells serve as the primary suppliers of IFN-alpha when exposed to viruses [57].

Additional confirmation about IFN role is provided by a study indicating that adults with AD encounter increased occurrences of viral or bacterial infections beyond the skin or at a systemic level [58].

The exposome hypothesis posits that the modern lifestyle’s environmental exposure to harmful substances can impact the epithelial barriers of the skin, gastrointestinal tract, and airways. These substances include, but are not limited to, cleaning products, detergents, microplastics, nanoparticles, elevated concentrations of ozone and particulate matter, cigarette smoke, and certain food additives such as enzymes and emulsifiers. Sustained exposure to microplastics at low doses holds the potential to induce dysfunction in the intestinal barrier and cause injury to epithelial cells [59,60]. Findings from a study on human lung epithelial cells indicate that exposure to microplastics, such as inhaled polystyrene, results in inflammatory and oxidative damage. Additionally, it leads to the breakdown of intercellular junction proteins in the lung, contributing to impaired lung barrier function [61].

The alternated skin barrier function is as we have already emphasized present in AD as well as alternated skin microbiome [62]. Individuals with AD commonly experience a reduction in bacterial diversity and an increased prevalence of the pathogenic bacterium S. aureus on their skin. Several factors contribute to this, including elevated skin pH, decreased levels of FLG and its associated breakdown products, and reduced levels of antimicrobial peptides in AD patients. These conditions create a conducive environment for the colonization of *S. aureus* on the skin, potentially triggering cutaneous inflammation and exacerbating AD symptoms through direct proteolytic damage to the epidermal barrier and immune dysregulation [63]. Colonization by S. aureus is linked to the severity and persistence of AD, as well as infectious and atopic comorbidities [62,64,65]. The activation of the immune system by S. aureus is facilitated through the expression of proteases, toxins, superantigens, and other virulence factors. Consequently, S. aureus skin colonization is hypothesized to coincide with the development of the atopic march. Supporting this, a recent experimental study demonstrated that an enterotoxin-producing S. aureus strain can induce allergen-induced excessive lung inflammation and airway hyperreactivity through an IL-17A-dependent mechanism, while also intensifying type 2 inflammatory responses [66].

Beyond disturbances in skin microbial communities, an increasing body of evidence highlights the significance of gastrointestinal dysbiosis in the initiation of AD and the broader atopic march. The gut microbiota plays a crucial role in influencing and modulating the immune response, thereby influencing susceptibility to immune-mediated disorders, including AD and allergic diseases [67,68].

Marenholz and colleagues conducted a genome-wide association study (GWAS) 2428 cases with AD in infancy and AA in childhood, along with 17,034 controls. Their investigation identified seven susceptible sites linked to the atopic march: FLG [1q21.3], AP5B1/OVOL1 [11q13.1], IL4/KIF3A [5q31.1], IKZF3 [17q21], C11orf30/LRRC329, EFHC1 [6p12.3], and rs99322 [12q21.3] [69]. Additionally, Gupta et al., conducted pathogenic genes: IL4, IL5, TSLP, RNASE3, FOXP3, KCNE4, CD4, IL4R, and CCL26. These genes were identified through large-scale and high-throughput bioinformatics analyses, and their roles in the atopic march await further experimental validation [70].

Epigenetic mechanism plays a role in governing gene expression and can be implicated as a causative factor in diseases. Numerous epigenome-wide studies have uncovered associations between DNA methylation in blood and conditions such as FA and AA [71,72,73].

Peng et al. have conducted DNA methylation analyses on cohorts from the Generation R study and Project Viva. Through meta-analyses they established associations between differential methylation profile in the peripheral blood of mid-childhood children and factors such as food allergens, environmental allergens, and atopic sensitization. Various genes linked to methylation sites were enriched in pathways related to AA, including eosinophil peroxidase, IL-4, IL-5 receptor A and proteoglycan 2. Additionally, the study identified several methylation site sin cord bloods that were correlated with allergic phenotypes in mid-childhood. Notably, some of these cord blood methylation sites persisted into mid-childhood, suggesting a longitudinal time trend [74]. The aforementioned findings imply that epigenetics could play a role in the development of allergic diseases [1].

## 5. Prevention Strategies for Atopic March/Atopic Multimorbidity Development

Since the compromised skin barrier function is thought to be an initial point of atopic multimorbidity development, one of the principal strategies in prevention is preservation of skin barrier function [75]. The first authors that have shown that regular application of standard emollients decreases skin pH and increases colonization of Streptococcus salivarius are Horimukai and Simpson [76,77]. The Prevention of AD By a Barrier Lipid Equilibrium Strategy (PEBBLES)study revealed that applying a trilipid-rich emollient, predominantly composed of ceramides, topically to high-risk infants from birth until six months of age reduces the likelihood of food sensitization at six to twelve months [78]. Notably, the introduction of the emollient cream within the first three weeks of life showed a significant impact [79]. The effectiveness of emollients in preventing AD has been called into question by two larger studies. One of them is The preventing of AD and Allergies in Children (PreventADALL) in which emollient use after twelve months did not reduce the incidence of AD but has mildly increased the risk of AD (3.1%, 95% CI: 0.3% to 6.5%). Ref. [80] The other study was the Barrier Enhancement for Eczema Prevention (BEEP) trial that followed high risk infants. At the age two years old, there were no significant difference in AD incidence and/or severity [81]. There is still a considerable amount to be clarified concerning the „best practices“ for the use of emollients. It remains uncertain whether emollients actually prevent AD and FA, or if they merely postpone their onset or reduce their severity. Additionally, there has been limited exploration into assessing the varying significance of different ingredients in emollients [82].

Supplementing with probiotics during pregnancy and/or infancy might offer protection against the onset of AD [83,84]. However, there is currently no evidence to support their protective effect against FA or other allergic disorders [85]. There is a lack of adequate evidence supporting the use of other nutritional interventions, including prebiotics, hydrolyzed formulas, or vitamin D supplementation [86].

Numerous randomized controlled trials have demonstrated introducing allergenic foods like peanut or egg to high-risk infants, especially those with severe AD or existing food sensitization, can potentially lower the risk of developing peanut or egg allergies, respectively [87,88,89].

Various primary prevention approaches for asthma and allergic rhinitis have been explored, such as early life house dust mite avoidance and prophylactic sublingual immunotherapy in sensitized children [90]. However, none of these strategies have demonstrated enough evidence to warrant their inclusion in routine clinical practice at present [91].

## 6. Biomarkers of the Atopic Multimorbidity

Advancements in technology, including transcriptomics, genomics, and proteomics, open new avenues for exploring potential biomarkers of atopic march and atopic multimorbidity [92]. The idea of biomarkers presents a key for future personal and precise treatment. So far, several predictors or biomarkers have been found. The expression of fatty-acid-binding protein 5 (FABP5) in the skin and T cells of individuals with atopic march and in murine models of atopic march showed a positive correlation with IL-17A levels in both the skin and serum. This suggests that FABP5 may play a role in the advancement of the atopic process by fostering Th17 inflammation. Furthermore, FABP5 is proposed as a potential biomarker for atopic march [93]. The variation in the Toll-like receptor 2 genes (TLR2-16934A > T) among individuals with AD and total IgE levels ≥ 106 IU/mL is linked to the presence of asthma, allergic conjunctivitis, or a family history of atopic diseases [94]. The Th17 pathway and the IL-17 cytokine family might play a role in the progression of allergic inflammation, with serum IL-17 levels correlating with the severity of allergic rhinitis in patients. Additionally, the Th17/IL-23 pathway is implicated in the development of asthma. IL-23 levels are significantly elevated in children with AA compared to healthy controls [95,96].

Davidson et al. have recently put forth recommendations for future research to explore potential biomarkers. These suggestions offer avenues for investigating the atopic march. The proposed strategies include: (1) examining protein, RNA, and lipid signatures in infants before and after AD using multi-omics approaches; (2) analyzing transcriptomics, proteomics, metabolomics, and the cell types of infant blood sequentially; (3) conducting sequential immune profiling of the blood, encompassing serology, cytokine profiles, and the evolution of specific B and T cells; (4) investigating the microbiomes in the skin and gut from birth; and (5) considering potential maternal delivery effects on atopy [2].

The strength of our review is that it provides a comprehensive overview of the atopic march outlining its historical context, evolution in understanding, and current state of knowledge. The manuscript critically assesses the initial perception of the atopic march as the sole trajectory of allergic disease development. It acknowledges the different/similar pathological mechanisms involved in the development of different atopic trajectories. The emphasis on portraying the atopic march alongside other patterns in the field of childhood atopic disease development reflects the relevance in the field. Given the complexity of the topic and the evolving nature of research of this field, the review may not cover every recent study or development. The scope limitation is inherent in reviews of this nature and should be considered when interpreting the findings.

## 7. Conclusions

The widely recognized phenomenon referred to as the “atopic march” manifests in only a subset of patients, despite the higher prevalence of the development of additional atopic diseases in individuals with atopic dermatitis. A more fitting term for this scenario is “atopic multimorbidity.” It is imperative to underscore the importance of accurately identifying patients experiencing the progression of atopic diseases. The foundational groundwork for researching potential biomarkers and preventive measures has been laid, necessitating further investigations to enhance the care of individuals with atopic multimorbidity.

## Data Availability

Not applicable.
